# *Mycobacterium mageritense* submandibular lymphadenitis in children: diagnostic challenges and the role of metagenomic next-generation sequencing: two case reports and literature review

**DOI:** 10.3389/fmed.2026.1936269

**Published:** 2026-10-07

**Authors:** Li Li, Zhijie Xing, Liyan Liu, Xiao Mu, Yunpeng Zhai, Shengwei Luan, Jian Li, Qingwei Guo

**Affiliations:** 1 Department of Infectious Diseases, Children's Hospital Affiliated to Shandong University, Jinan, Shandong, China; 2 Department of Hematology and Oncology, Children's Hospital Affiliated to Shandong University, Jinan, Shandong, China; 3 Department of Thoracic Oncology, Children's Hospital Affiliated to Shandong University, Jinan, Shandong, China; 4 Department of Ultrasound, Children's Hospital Affiliated to Shandong University, Jinan, Shandong, China

**Keywords:** *mycobacterium mageritense*, next-generation sequencing (mNGS), non-tuberculous mycobacterium, rapidly growing mycobacterium, submandibular lymphadenitis

## Abstract

*Mycobacterium mageritense* is an extremely rare, rapidly growing nontuberculous mycobacterium (NTM). Pediatric cases are rarely reported worldwide, and the non-specific clinical manifestations, low sensitivity of conventional microbiological tests, and limited specimen volume from children often lead to diagnostic delays. We describe the clinical data of two immunocompetent children with submandibular lymphadenitis caused by *Mycobacterium mageritense*, both diagnosed by metagenomic next-generation sequencing (mNGS). We also systematically reviewed the relevant literature and performed a pooled analysis of 36 eligible cases (the two new paediatric cases from the present study included). Case 1 involved a 6-year-old girl with dental caries and acute fever who was initially misdiagnosed with common bacterial infection and received ineffective empirical antibiotics for over two weeks; mNGS of aspirated pus confirmed the diagnosis. Case 2 involved a 9-year-old girl with dental caries who presented only with submandibular lymphadenopathy and normal laboratory findings, with a diagnostic delay of up to two months; mNGS of lymph node tissue identified the pathogen. Literature-pooled analysis of 36 patients revealed a mean diagnostic delay of 4.69 months (median 2.00 months, IQR: 0.81–5.75 months). Conventional culture had low sensitivity, often requiring prolonged incubation (≥96 h) or special media to obtain positive results. Initial misdiagnosis occurred in 17.6% of cases. Patients diagnosed by NGS achieved more rapid and accurate pathogen identification. This report suggests that mNGS, as a culture-independent, unbiased pan-pathogen detection technique, shows clear advantages in identifying rare and fastidious pathogens such as M. mageritense. For pediatric patients with dental caries, fever, and cervical lymphadenopathy unresponsive to conventional therapy, clinicians should suspect *Mycobacterium mageritense* infection, and mNGS is an effective tool for its rapid and accurate diagnosis.

## Introduction

*Mycobacterium mageritense* is a rapidly growing non-tuberculous mycobacterium (RGM) first described in 1997, named after “Magerit,” the old Arabic name of Madrid, Spain, where the initial isolates were obtained (1). This environmental organism is widely distributed in water and soil and has been increasingly recognized as a cause of clinical infections, including skin and soft tissue infections, surgical site infections, catheter-related bloodstream infections, and pulmonary disease (2–6). *Mycobacterium mageritense* infections have traditionally been associated with immunocompromised individuals; however, recent reports have also documented cases in immunocompetent hosts. Most of these occur in adults, and pediatric cases remain exceedingly rare (7–9).

Diagnosing *Mycobacterium mageritense* infection presents multiple challenges. First, its clinical manifestations are non-specific and overlap with common bacterial infections (9). Second, conventional bacterial cultures have low sensitivity for this organism; it grows slowly on routine media and may be overlooked or misidentified as diphtheroid contaminants (10). Third, owing to the organism's fastidious growth requirements and the inherently small specimen volumes obtainable from children, achieving a definitive diagnosis is particularly difficult in pediatric cases.

Metagenomic next-generation sequencing (mNGS) has emerged as a powerful tool for diagnosing rare and fastidious infections, especially when conventional tests are negative or sample volumes are limited (11, 12).In this report, we describe two pediatric cases of *Mycobacterium mageritense* lymphadenitis diagnosed by mNGS and systematically review the published literature, comparing disease characteristics between children and adults, with a focus on diagnostic delay and the role of mNGS.

### Case presentation

#### Case 1

A 6-year-old girl with a history of dental caries presented with toothache for 11 days and fever for 9 days. Prior to admission, she had undergone a pulpotomy at a dental hospital and received a 5-day course of cefazolin and azithromycin. Despite this treatment, her toothache and fever persisted, accompanied by progressive swelling of the left side of the face and a tender, enlarging mass in the left submandibular region. She had no cough, vomiting, diarrhea, headache, rash, night sweats, or joint swelling. Physical examination revealed left-sided facial swelling. A hard, tender 1.5  ×  1.0 cm mass was palpable in the left mandibular area.

Laboratory evaluation **(**Table 1**)** revealed leukocytosis with neutrophilia, elevated C-reactive protein, and high levels of erythrocyte sedimentation rate. Procalcitonin, immunoglobulin levels, tuberculosis interferon-gamma release assay (IGRA), and PPD test were normal. Chest computed tomography (CT) showed no abnormalities. Neck ultrasound (Figure 1A) demonstrated a 1.7 × 1.0 × 1.0 cm hypoechoic mass in the left submandibular region with clear borders, heterogeneous internal echoes, and patchy anechoic areas suggesting liquefaction.

**Table 1:** Laboratory findings of two pediatric patients with *mycobacterium mageritense* lymphadenitis.

Test	Case 1	Case 2	Reference range
WBC Count (× 10⁹/L)	12.09	8.07	4.0–12.0
Neutrophils (absolute) (× 10⁹/L)	6.23	3.97	1.5–8.0
CRP (mg/L)	83.97	<3.22	<5.0
PCT (ng/mL)	0.05	0.05	<0.10
ESR (mm/h)	49	4	<15
IgG (g/L)	11.0	9.1	5.9–14.3
IgM (g/L)	1.94	1.21	0.45–2.08
IgA (g/L)	1.18	1.11	0.38–2.22
CD3+ T cells (%)	70	70	59.5–75.6
CD3 + CD4+ T cells (%)	37	41	28.5–41.1
CD3 + CD8+ T cells (%)	25	26	19.7–32.0
CD4+/CD8 + ratio	1.52	1.36	1.02–2.05
PPD skin test	Negative	Negative	Negative
IGRA(IU/mL)	0	1	<5
Blood culture	Negative	Negative	Negative

CRP, C-reactive protein; PCT, procalcitonin; ESR, erythrocyte sedimentation rate; IGRA, interferon-gamma release assay; PPD, purified protein derivative.

**Figure 1:**
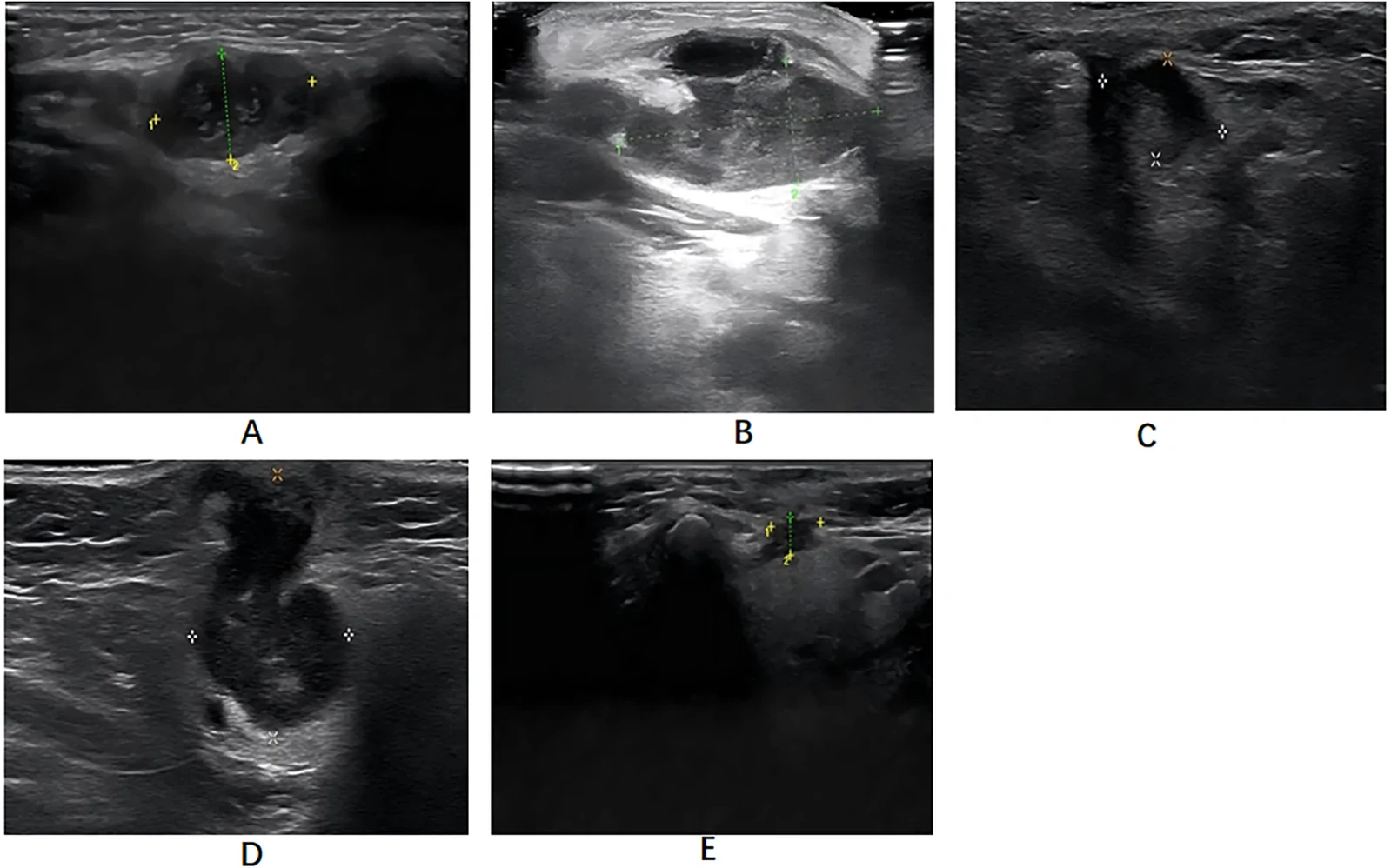
Serial neck ultrasound images. **(A)** Admission ultrasound of Case 1, showing an enlarged submandibular lymph node. **(B)** Two-week follow-up ultrasound of Case 1, demonstrating enlargement and liquefaction. **(C)** Three-month follow-up ultrasound of Case 1, showing complete resolution. **(D)** Admission ultrasound of Case 2, revealing a hypoechoic mass. **(E)** Three-month follow-up ultrasound of Case 2, showing complete resolution.

The patient was initially diagnosed with suppurative lymphadenitis and periapical abscess. She received 14 days of intravenous treatment with meropenem and metronidazole, and underwent root canal therapy. Although the fever and facial swelling subsided, the submandibular mass gradually increased in size and became more painful. Ultrasound examination revealed a 2.9 × 1.9 × 1.5 cm lesion containing pus (Figure 1B).

Subsequently, we performed ultrasound-guided aspiration of purulent fluid for metagenomic next-generation sequencing (mNGS) and routine culture, and obtained a tissue biopsy for histopathological examination. mNGS identified 131 sequence reads of *Mycobacterium mageritense* with 99% confidence score (Table 2). Initial routine bacterial culture showed no growth at 5 days. After the metagenomic next-generation sequencing (mNGS) results indicated *Mycobacterium mageritense*, we extended the culture incubation period to 10 days, which ultimately confirmed the presence of the bacterium. Owing to the limited number of colonies, only the disk diffusion method was feasible for antimicrobial susceptibility testing; the inhibition zone for azithromycin was small, whereas those for linezolid and amikacin were larger. Histopathological examination showed granulomatous lymphadenitis, characterized by multiple epithelioid granulomas, scattered multinucleated giant cells, focal necrosis **(**Figure 2A); Acid-fast bacilli (AFB) stain negative **(**Figure 2B**).**

**Table 2:** mNGS results of the two patients.

Sample	Genus				Species		
	Type	Name	Relative abundance^a^	mNGS reads^b^	Name	Confidence score^c^	mNGS reads
Case 1	G+	Mycobacterium	68.5%	297	*Mycobacterium mageritense*	99%	131
Case 2	G+	Mycobacterium	5.1%	24	*Mycobacterium mageritense*	99%	14

a The proportion of the microorganism in all the microorganisms detected.

b The mNGS reads of the microorganism detected at the genus/species level in rigid alignment.

c Confidence score is the species-level classification score generated; a score ≥99 indicates high-confidence species identification.

**Figure 2:**
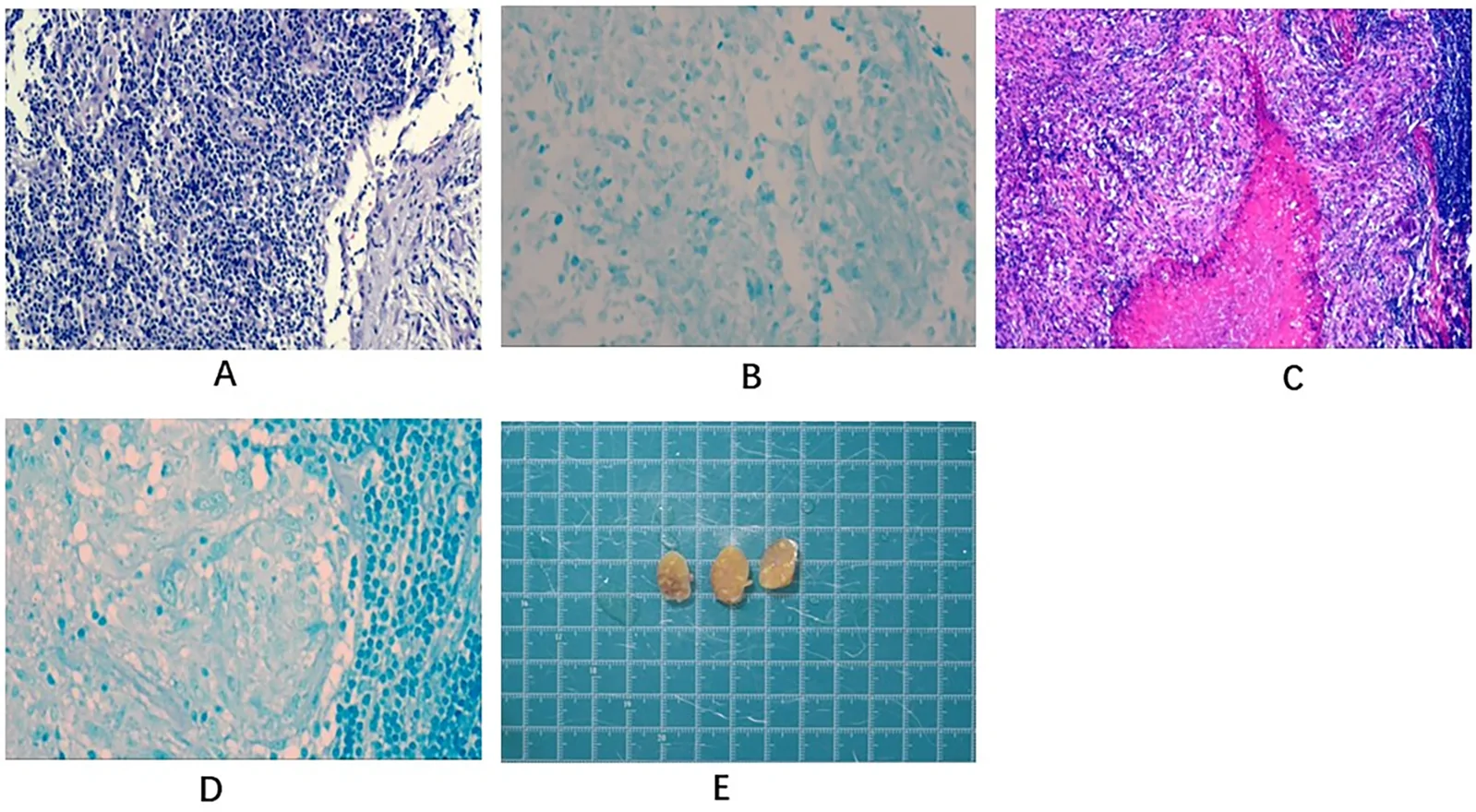
Histopathological and surgical specimen findings of lymph-node biopsies. **(A)** Granulomatous lymphadenitis in Case 1 (hematoxylin and eosin stain, original magnification ×100). **(B)** Acid-fast bacilli (AFB) stain negative in Case 1. (original magnification ×200). **(C)** Necrotizing granulomatous inflammation in Case 2 (hematoxylin and eosin stain, original magnification ×100). **(D)** Acid-fast bacilli (AFB) stain negative in Case 2. (original magnification ×200), **(E)** Surgical specimen of Case 2. (scale ba*r* = 1 cm).

Based on guidelines for the diagnosis and treatment of non-tuberculous mycobacterial disease in China, the patient started triple antimicrobial therapy consisting of linezolid, amikacin, and clarithromycin. During this period, we performed incision and drainage of the mass. The triple regimen continued for one month. Following clinical stabilization, the patient received an additional three months of dual therapy with linezolid and clarithromycin, resulting in complete resolution of the mass (Figure 1C). The patient achieved clinical cure and was discharged, but was subsequently lost to follow-up.

#### Case 2

A previously healthy 9-year-old girl presented with a gradually enlarging, mildly painful left submandibular mass over 2 months. She had no fever, cough, vomiting, diarrhea, rash, weight loss, or night sweats. Her medical history was notable for dental caries and gum bleeding. Prior to admission, she had received 11 days of empirical antibiotic therapy at another hospital, without significant clinical improvement. Physical examination revealed a firm, tender mass in the left submandibular region.

Laboratory investigations (Table 1), including white blood cell count, C-reactive protein (CRP), procalcitonin, erythrocyte sedimentation rate (ESR), and liver function tests, were all within normal limits. Tuberculosis interferon-gamma release assay (IGRA), PPD skin test, and chest computed tomography (CT) were negative. Neck ultrasound **(**Figure 1D) demonstrated a 1.7 × 1.4 × 1.3 cm hypoechoic mass in the left submandibular region with clear borders, irregular shape, and heterogeneous internal echoes. A 0.5 × 0.4 cm anechoic area within the mass suggested liquefaction.

Given the very limited volume of drainable pus, we performed only an ultrasound-guided core needle biopsy of the lymph node and submitted tissue specimens for histopathological examination and mNGS. Histopathological examination (Figure 2C) revealed necrotizing granulomatous inflammation characterized by central caseous-like necrosis, epithelioid-cell granulomas, and Langhans-type giant cells, while AFB staining yielded negative results (Figure 2D). The gross appearance of the surgical specimen from Case 2 is presented in Figure 2E. Metagenomic next-generation sequencing (mNGS) detected *Mycobacterium mageritense* with 14 sequence reads at 99% classification confidence score (Table 2).

The patient started a triple regimen consisting of linezolid, amikacin, and clarithromycin. After one week of initial therapy, we performed surgical intervention. The triple regimen continued for a total of two months, followed by an additional one month of oral dual therapy with linezolid and clarithromycin. At the end of treatment, the cervical mass completely resolved, and the patient achieved a favorable outcome (Figure 1E).

## Materials and methods

### Metagenomic next-generation sequencing

Specimens were collected from the patients: 0.8 mL pus sample for Case 1 and 1 g lymph-node tissue for Case 2. DNA was extracted from specimens with the QIAamp DNA Mini Kit (Qiagen, Germany) following the manufacturer's protocol. Differential lysis was employed for host depletion, with only a negative control (sterile water) included. DNA libraries were constructed with the KAPA HyperPrep Kit (KAPA Biosystems, USA) and sequenced on an Illumina NextSeq 550 platform. Case 1 generated approximately 12.5 million raw reads and 8.2 million clean reads, whereas Case 2 yielded 10.8 million raw reads and 7.1 million clean reads. Host-derived reads accounted for 92%–94% of total clean reads. *Mycobacterium mageritense* specific reads were 131 (RPM = 15.9) for Case 1 and 14 (RPM = 1.97) for Case 2, respectively. A negative control sample was processed and sequenced in parallel in each sequencing run for quality control.

For bioinformatic analysis, Trimmomatic and fastp were used for raw-read quality filtering. The Burrows–Wheeler Aligner was applied to remove human host sequences. Taxonomic classification of sequencing reads was performed using Kraken2 (v2.1.3) with default parameters including –confidence 0.2 and –threads 40. Only sequencing reads showing ≥96% nucleotide identity against reference genomes were retained, and multi-locus ambiguous reads mapping to multiple positions within the same genus were excluded. A confident pathogen identification required composite criteria: ≥3 unique target reads, the pathogen ranked among the top10 microbial taxa, and complete absence of corresponding reads in negative controls.

### Literature search strategy

This literature search strategy was performed in accordance with the PRISMA 2020 reporting guidelines. We conducted a comprehensive literature search in the PubMed databases using the keywords “*Mycobacterium mageritense*” or “*Mycolicibacterium mageritense*” up to January 2026. And a secondary search through references was also performed. Eligible articles had to contain extractable complete individual-level clinical data with no age restrictions. The following exclusion criteria were adopted (1) literature focusing on other nontuberculous mycobacteria that only briefly mention *Mycobacterium mageritense* or *Mycolicibacterium mageritens*e; (2)animal experiments, *in-vitro* studies, editorials, narrative reviews, and commentaries; (3) literature without accessible clinical patient data. Ultimately, a ﬂowchart of the literature screening process was summarized in Figure 3.

**Figure 3:**
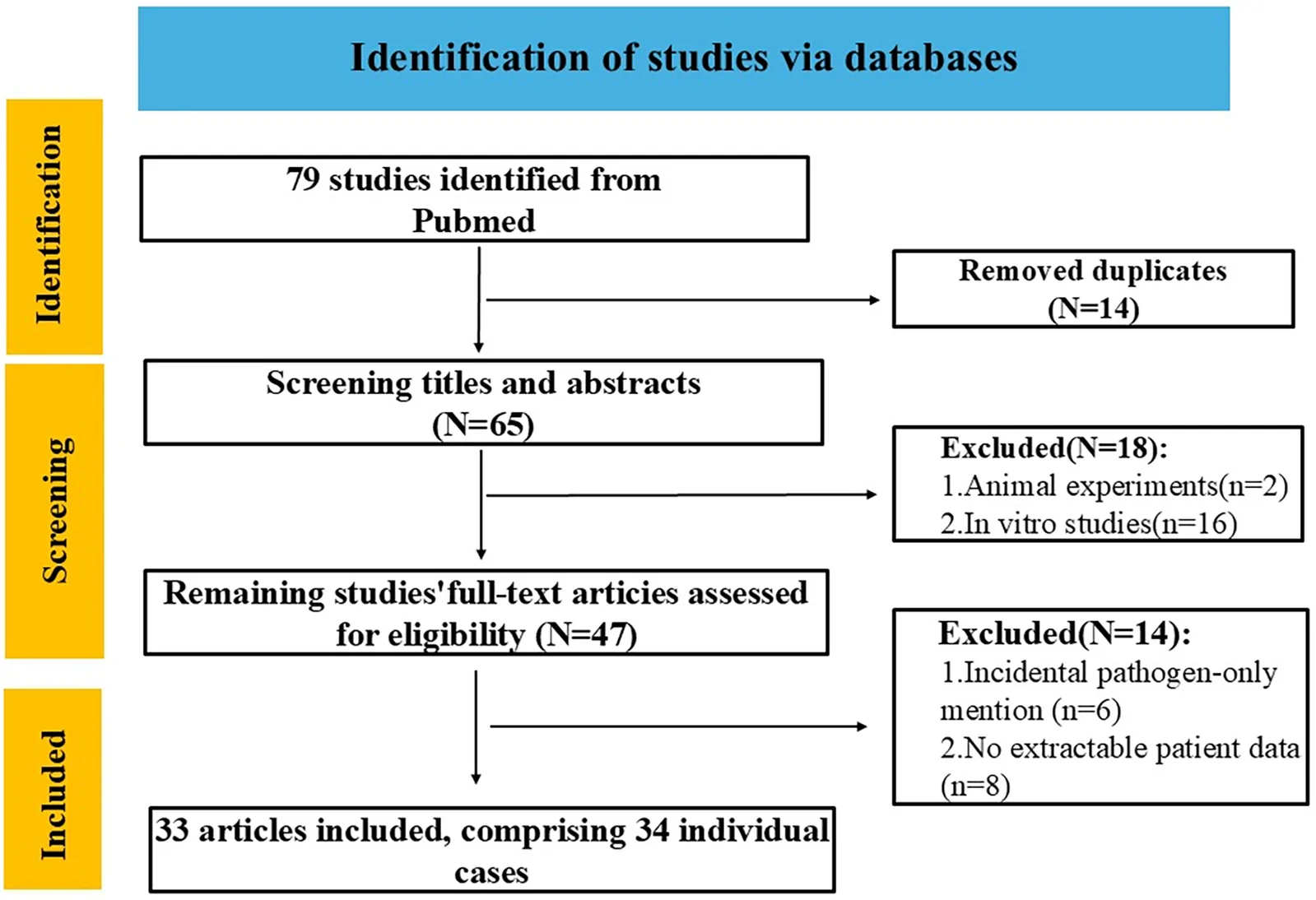
PRISMA 2020 flow-diagram of literature screening process.

## Discussion and literature review

We report two immunocompetent children with submandibular lymphadenitis caused by *Mycobacterium mageritense*, diagnosed by metagenomic next-generation sequencing (mNGS). This study is the first to propose dental caries as a potential portal of entry for *Mycobacterium mageritense* cervical lymphadenitis in children, and also the first case series of paediatric *Mycobacterium mageritense* infection diagnosed by mNGS.

*Mycobacterium mageritense* was first described in 1997 (1), but pediatric cases remain rare (7, 9), Our systematic review of 34 published cases worldwide identified only 2 previously reported pediatric cases (Supplementary Table S1; references 22–41). The 2 new pediatric cases described in this study double the number of reported pediatric cases.

Among the 34 literature cases, female patients were markedly more numerous than males with a male-to-female ratio of approximately 1:2.4. Patients aged 41 to 60 years accounted for the largest proportion at 50.0%, while pediatric cases were rare, accounting for only 5.9% (2/34). In terms of underlying conditions, 20 patients suffered from chronic diseases or had a history of tumor surgery, ranking the highest. There were 8 cases related to surgical procedures, 4 cases associated with invasive operations and acute illnesses, 4 cases with external high-risk exposure history, and 3 healthy patients without underlying diseases. Infectious sites were diverse. Skin and superficial soft tissue infection was the most common, occurring in 16 cases. Five cases catheter-related infection and four cases involving cardiovascular devices. Pulmonary and pleural infection was noted in 3 cases, and abdominopelvic visceral infection in 2 cases. Single cases of prosthetic joint, central nervous system and ocular infection were also observed respectively.

Diagnostic delay is a hallmark of *Mycobacterium mageritense* infection. To compare diagnostic latency between pediatric and adult patients, we extracted unified monthly delay data from 36 eligible cases. In our systematic review, the mean interval from symptom onset to definitive diagnosis was 4.69 months (median 2.00 months, IQR: 0.81–5.75 months) overall. Subgroup analysis revealed substantially shorter delays in pediatric patients than in adults: among the four pediatric cases, the mean diagnostic delay was 1.35months (median 1.33months, IQR: 0.58–2.11months), whereas in 32 adult cases, the mean delay was 5.11months (median 2.25months, IQR: 1.0–6.50months).

Two major factors contribute to this substantial delay. First, clinical manifestations are nonspecific, leading to frequent misdiagnosis as common bacterial infections or tuberculosis (6, 13). Subsequent empirical antibiotic therapy of variable duration further contributes to substantial delays in definitive diagnosis. Six cases (17.6%) were mistakenly diagnosed as infections caused by other pathogens such as *Corynebacterium* or *Nocardia* (2 cases), *Dermatophilus congolensis* (1 case), *M. abscessus* (1 case), *M. fortuitum* (1 case), *M. smegmatis* (1 case). Of the 34 patients, 32 received empirical antimicrobial therapy, including 16 treated for more than two weeks and 6 for longer than three months. Second, conventional microbiological tests demonstrate low sensitivity. Although *Mycobacterium mageritense* can grow on standard blood agar within 3–5 days, its Gram-positive bacillary morphology is easily misidentified as *Corynebacterium* or *Nocardia* species (9, 10). More importantly, routine clinical bacterial cultures are typically incubated for only 48 h, which readily results in false-negative outcomes. In addition, this organism is vulnerable to decontamination procedures routinely used for mycobacterial culture, further contributing to negative culture results (14, 15). Among all cases, positive strains were only obtained after prolonged incubation of no less than 96 h in 7 cases (20.6%), and special culture media were required for detection in 10 cases (29.4%). Due to the low clinical incidence and stringent culture requirements of this strain, 28 cases (82.4%) required combined culture and molecular techniques.

In this context mNGS exhibits unique and irreplaceable diagnostic value. As an unbiased, hypothesis-free approach, mNGS directly extracts nucleic acids from clinical specimens for high-throughput sequencing, bypassing the critical bottleneck of microbial culture. It is therefore especially valuable for detecting fastidious, rare, or post-antibiotic infections (12, 15). In both of our patients, mNGS rapidly and definitively identified *Mycobacterium mageritense*, providing crucial guidance for timely targeted treatment. Even though only 14 sequence reads were detected in the second patient, a conclusive diagnosis was achieved by integrating typical histopathologic changes and clinical manifestations, fully illustrating the high sensitivity of mNGS in low-biomass specimens. Compared with traditional molecular methods such as 16S rRNA or rpoB gene sequencing, mNGS requires no prior pathogen suspicion and enables panoramic pathogen detection in a single test, conferring a distinct advantage in cases with coinfection or atypical presentations (12, 15).

Despite the improved diagnostic yield brought by mNGS, the exact invasion route of pediatric *Mycobacterium mageritense*-associated cervicofacial lymphadenitis remains incompletely elucidated. In immunocompetent children, disruption of cutaneous or local mucosal barriers may serve as an important prerequisite for NTM invasion into human tissues (16). Specifically, dental caries and necrotic dental tissue may act as a portal of entry for environmentally-derived rapidly-growing nontuberculous mycobacteria (17). Previous case reports have described odontogenic granulomatous lesions and subsequent submandibular swelling triggered by NTM in the setting of dental disease or dental procedures. The putative underlying mechanism involves breakdown of the oral mucosal barrier secondary to dental caries, gingival bleeding, or endodontic manipulation, which enables environmental mycobacteria to invade host tissues and subsequently disseminate via lymphatic routes to regional lymph nodes, resulting in opportunistic infection (16, 18, 19). Both children in our cohort had dental caries, and their lymphadenitis was strictly localized to the ipsilateral submandibular region, providing further clinical support for this hypothesis.

To facilitate rapid clinical identification and standardized management of high-risk children, we summarized a concise diagnostic algorithm for pediatric *Mycobacterium mageritense* submandibular lymphadenitis based on our clinical experience and pooled literature data (Figure 4). For immunocompetent children with dental caries and refractory unilateral cervical lymphadenopathy unresponsive to conventional antibiotics, timely mNG detection of lesion specimens is recommended as the preferred early diagnostic tool, avoiding prolonged ineffective empirical antibacterial therapy and shortening diagnostic delay. The flowchart integrates dental risk screening, pathogen detection strategy and targeted treatment initiation, providing practical bedside guidance for pediatric infectious disease clinicians.

**Figure 4:**
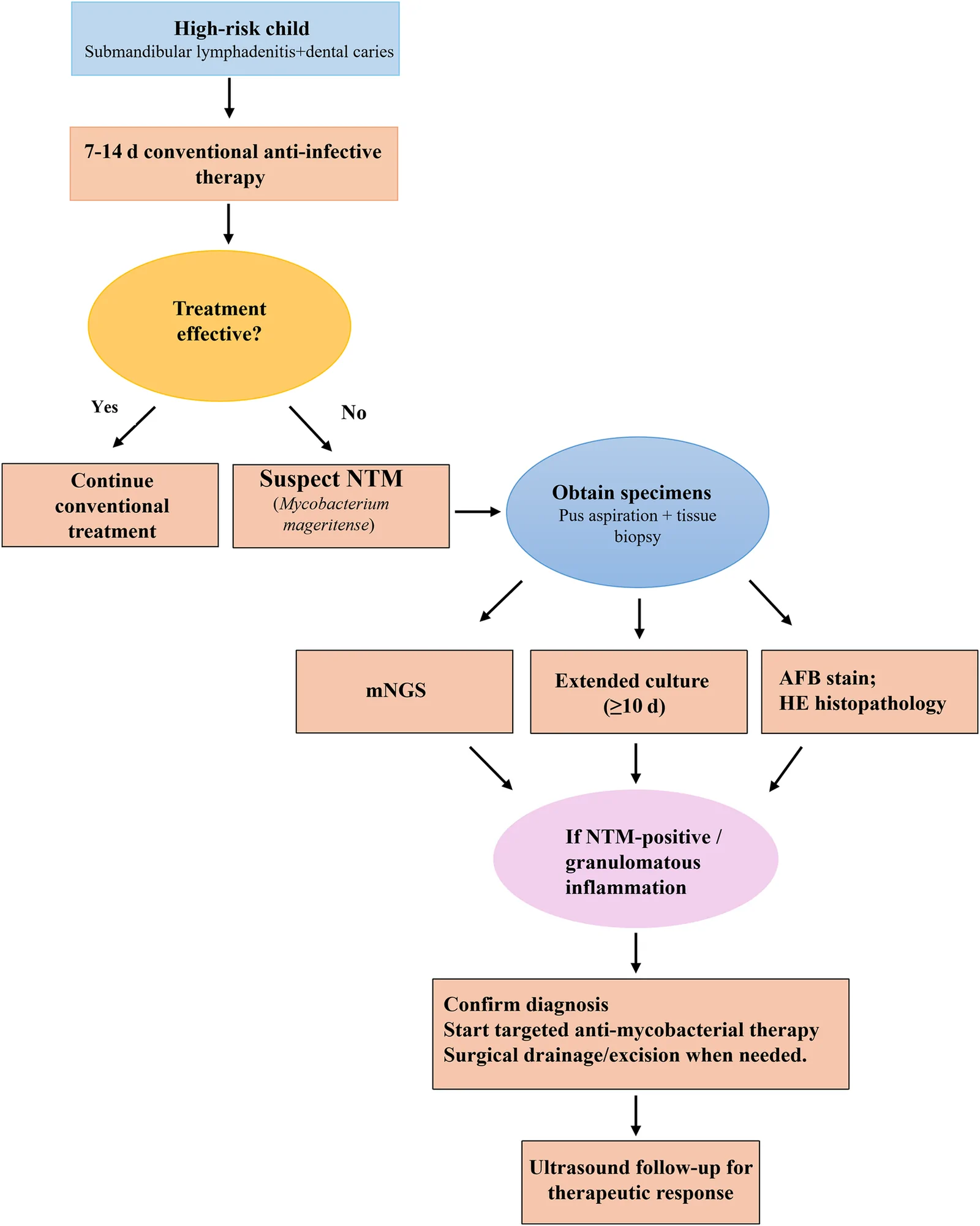
Diagnostic algorithm for suspected *mycobacterium mageritense* submandibular lymphadenitis in high-risk children.

Treatment of *Mycobacterium mageritense* infection remains challenging. This pathogen intrinsically harbors the erm(40) gene, conferring high-level resistance to macrolides (e.g., clarithromycin and azithromycin), which are often used as empirical therapy for nontuberculous mycobacterial infections (20, 21). Therefore, accurate and rapid species identification is essential to avoid ineffective treatment. According to the literature, fluoroquinolones (e.g., levofloxacin, moxifloxacin), linezolid, amikacin, and imipenem show relatively good *in vitro* activity (8, 20). However, antimicrobial options are significantly more limited in pediatric patients than in adults (7, 8). In our initial regimen, we employed linezolid plus amikacin and clarithromycin, which achieved clinical efficacy despite *in vitro* Macrolides antibiotics resistance. This outcome was likely attributable to synergistic drug effects combined with adequate surgical debridement and drainage. Collectively, these findings underscore that the management of rapidly growing mycobacterial infections should be individualized, taking into account both susceptibility profiles and clinical response, and guided by multidisciplinary input rather than relying solely on *in vitro* test results.

## Study limitations

This study describes two rare cases of *Mycobacterium mageritense* submandibular lymphadenitis in immunocompetent children, expanding current clinical knowledge. We hypothesize that dental caries may serve as a potential portal of entry for this pathogen, which offers valuable clues for bedside clinical screening. Nevertheless, several limitations of the present work should be acknowledged. First, although the hypothesis regarding dental caries as an infectious entry portal is partially supported by published literature and our two institutional cases, it still requires validation by large-scale multicenter pediatric cohort studies. Second, owing to the limited volume of clinical specimens obtained from children and extremely low bacterial colony yields, complete quantitative minimum inhibitory concentration (MIC) antimicrobial susceptibility data were unavailable in our series.

## Conclusion

*Mycobacterium mageritense* is a rare but important pathogen of paediatric lymphadenitis. Diagnosis is often delayed. mNGS is an effective tool to overcome these diagnostic obstacles and achieve rapid, accurate diagnosis. Clinicians should increase awareness of atypical mycobacteria, particularly *Mycobacterium mageritense*, in children.

## Data Availability

The original contributions presented in the study are included in the article/Supplementary Material, further inquiries can be directed to the corresponding author.
