# Supplementary material for: Mycobacterium mageritense submandibular lymphadenitis in children: diagnostic challenges and the role of metagenomic next-generation sequencing: two case reports and literature review
Source: Front Med (Lausanne). 2026 Oct 7;13:1936269. doi: 10.3389/fmed.2026.1936269 (PMC13683177; doi:10.3389/fmed.2026.1936269)
Supplement: Supplementary file 1 [file Table1.docx]

Supplementary Table 1. Summary of reported cases of *Mycobacterium mageritense* infection

| References | Sex | Age | Underlying conditions / risk factors | Infection site | Time from onset to diagnosis (months) | Diagnostic methods | Empirical antibiotic duration | Treatment regimen & duration | Outcome | Complications |  |
| --- | --- | --- | --- | --- | --- | --- | --- | --- | --- | --- | --- |
| Fukushima et al., 2026^(8)^ | M | 48 | Healthy (immunocompetent) | Lower limb cutaneous bscess + ipsilateral inguinal lymphadenitis | ~1.4 months | MGIT culture (10d), MALDI‑TOF MS, whole‑genome sequencing | Cefdinir + cefaclor (~10d) | TMP‑SMX + minocycline (13d) → linezolid + levofloxacin → levofloxacin + minocycline (4 months); total 6 months) | Cured | Linezolid‑induced pancytopenia |  |
| Frings et al., 2025^(22)^ | F | 46 | Previously healthy; cleaned aquarium (environmental exposure) | Skin (face, dorsum of hands,mandible), cervical lymph nodes | ~0.8–1.0 months | Gram stain, Ziehl‑Neelsen stain, culture, 16S rRNA PCR and sequencing (mixed M. wolinskyi + M. mageritense) | IV cefuroxime (several days to 2 weeks, ineffective) | Incision & drainage + IV amikacin + oral linezolid + doxycycline + moxifloxacin (12 weeks), then oral linezolid + doxycycline + moxifloxacin (3 months); total ~6 months | Cured | None |  |
| Fukumori et al., 2025^(8)^ | M | 70 | CKD, post‑TURBT for bladder cancer, gemcitabine + carboplatin | Prostatic abscess | 3 months  post‑TURBT; diagnosed on day 13 of admission | Gram stain, Ziehl‑Neelsen stain, blood agar culture (1d), MALDI‑TOF MS, 16S rRNA/hsp65/sodA sequencing | Tazobactam/piperacillin (5d) → cefepime (6d) | Drainage + imipenem/cilastatin + levofloxacin (2 months IV) → levofloxacin + doxycycline (10 months oral); radical cystectomy | Cured | Linezolid: anemia, visual impairment, lower limb numbness; doxycycline: neurological side effects |  |
| Ishikawa et al., 2025^(14)^ | M | 78 | ESRD (diabetic nephropathy), previous PD catheter‑related infections | PD exit‑site infection → PD peritonitis | ~5 months | Blood agar culture, MALDI‑TOF MS, 16S rRNA sequencing | Faropenem for 11 d before admission (multiple previous antibiotics) | Multiple regimens failed, finally sitafloxacin + TMP‑SMX for 18 months heter removal | Cured | None |  |
| Sakurai1 et al., 2025^(23)^ | F | 58 | IgA nephropathy, post‑kidney transplant, immunosuppressed (tacrolimus + MMF + prednisone), previous PD | Abdominal wall (site of removed PD catheter) | ~0.5–0.8 months | Gram stain (negative), routine culture (negative), Ziehl‑Neelsen stain (AFB positive), LAMP PCR | Ampicillin + drainage (ineffective); vancomycin (several days to 1 week) | Debridement; linezolid + levofloxacin (stopped linezolid due to pancytopenia), then levofloxacin alone for 2 months; MMF reduced | Cured (one recurrence, then successful, no relapse 6 months after stopping antibiotics) | None |  |
| Suzuki et al., 2025^(3)^ | F | 51 | Left breast cancer, post‑DIEP flap reconstruction; no comorbidities | Abdomen (umbilical and abdominal incisions) | ~0.5–0.8 months | Culture, acid‑fast stain | Not specified (initial local irrigation) | Multiple debridements + NPWT; antibiotics: imipenem + levofloxacin (IV) → linezolid + levofloxacin (oral) → doxycycline; total 4 months | Cured | Splenectomy (sequelae) |  |
| Takura et al., 2025^(24)^ | M | 54 | Esophageal cancer, salvage esophagectomy after chemoradiotherapy | Multiple abscesses (retrotracheal, abdominal wall, etc.) | ~0.5 months | CT, Ziehl‑Neelsen stain, mycobacterial culture, susceptibility testing | Broad‑spectrum antibiotics (several weeks) | According to susceptibility: stopped amikacin and imipenem/cilastatin, continued linezolid + levofloxacin; total duration not specified | Cured | None |  |
| Akazawa et al., 2024^(25)^ | F | 49 | Right breast cancer, post‑TRAM flap | Right breast, abdominal wall, periumbilical | ~1–2 months | Gram stain (weakly positive rods), sheep blood agar (96h white colonies), Ziehl‑Neelsen, MALDI‑TOF MS, susceptibility | Cephalexin (2 months ) | Repeated debridement + drainage; levofloxacin + TMP‑SMX (rash → stopped) → levofloxacin + doxycycline; total 6 months | Cured | None |  |
| Hu et al., 2023^(26)^ | M | 55 | Hypertension, diabetes, gout, on PD for 2 years | PD‑associated peritonitis | ~0.8 months | Mycobacterial culture (10d) | Cefazolin + ceftazidime (~2 weeks) | Linezolid + amikacin + imipenem → add azithromycin → catheter removal → switch to moxifloxacin; total ≥4 months | Cured | None |  |
| Alhariri etal.,  2023^(27)^ | F | 62 | Pacemaker implanted 3 years, prior pericarditis | Left chest wall (pacemaker generator site) | >12 months (abscess 12 mo post-implantation, recurred 3 mo after drainage, diagnosed >12 mo later) | Gram stain, acid‑fast stain (positive), mycobacterial culture, susceptibility (Mayo Clinic) | Not specified (no prior effective treatment) | Initial doxycycline + clarithromycin + levofloxacin (oral) → switched to moxifloxacin monotherapy (8 months) based on susceptibility; later generator + lead removal, intraoperative imipenem 1 week, new device implanted | Cured | None |  |
| Etesami et al., 2022 (patient 1)^(28)^ | F | 25 | Healthy; cryopreserved autologous facial fat injection (non‑sterile) | Face (cheeks, chin, oral mucosa) | ~1.5–2 months | Ultrasound, pus smear (AFB positive), PCR, DNA sequencing, histopathology (granulomatous) | Outside: vancomycin + imipenem + metronidazole (10d); our hospital: clindamycin + cefepime + acyclovir + levofloxacin + clarithromycin + doxycycline (ineffective); prednisolone 10d ineffective | Minocycline monotherapy, 2 months | Cured | None |  |
| Etesami et al., 2022 (patient 2)^(28)^ | F | 27 | Healthy; same clinic, cryopreserved fat injection | Face (multiple nodules, nasolabial fold, eyebrows, zygomatic arches) | ~1.5–2 months | Same as above | Outside: ciprofloxacin, cefazolin, cloxacillin, cephalexin, doxycycline (ineffective); our hospital: same empirical regimen as patient 1 + prednisolone 10d ineffective | Minocycline monotherapy, 2 months | Cured | None |  |
| García‑Boyano et al., 2022^(9)^ | M | 2y9m | Healthy | Right submandibular lymphadenitis | ~0.5–0.8 months | Liquid culture (6d), GenoType CM v2.0, MALDI‑TOF MS (2.4), WGS, microdilution susceptibility | Clarithromycin + ciprofloxacin (~3 weeks until susceptibility results) | Clarithromycin + ciprofloxacin (3 weeks) → linezolid (intolerant) → back to clarithromycin monotherapy, total 11 weeks; later surgical excision of residual node | Cured | None |  |
| Maheshwari et al., 2022^(29)^ | F | 42 | Laparoscopic hysterectomy 2 months earlier; immunocompetent | Right eye (choroiditis), bloodstream | ~1.5 months (visual loss 6 wk post-op; repeatedly positive blood cultures after several weeks) | MALDI‑TOF MS, susceptibility; initially misdiagnosed as septic embolism, retinal abscess | LMWH, parenteral antibiotics (several weeks, ineffective) | IV amikacin (stopped due to liver dysfunction) → oral meropenem + ciprofloxacin + TMP‑SMX; total 2 months | Improved (vision 2/60 → 6/60, healed choroiditis) | None |  |
| Sando et al., 2022^(2)^ | F | 69 | Untreated rheumatoid arthritis | Left cheek skin ulcer | Several months (after tennis ball bruise; confirmed within days of pustules) | MALDI‑TOF MS, WGS, susceptibility | Topical and oral antifungals (ineffective) | Levofloxacin + clarithromycin (12 weeks) | Cured | None |  |
| Ando et al., 2021^(30)^ | F | 85 | After pedicled rectus abdominis flap, huge ventral hernia repair (mesh) | Abdominal wall mesh infection | ~0.7 months | Culture (initially reported M. abscessus, corrected after 6 days) | Perioperative ceftriaxone; IV amikacin + imipenem + clarithromycin (6 days) | NPWT 4 weeks + IV amikacin/imipenem/clarithromycin (6 days) then levofloxacin, followed by oral levofloxacin + minocycline ~6 months | Cured | Aspiration pneumonia, uncontrolled pleural effusion |  |
| Koyama et al., 2021^(31)^ | F | 44 | Breast cancer, nivolumab, central venous port | Catheter‑related bloodstream infection, skin infection | 5 months | MALDI‑TOF MS, 16S rRNA sequencing | Not specified (started amikacin + meropenem + ciprofloxacin) | Debridement + catheter removal + amikacin + meropenem + ciprofloxacin (4 weeks)→ recurrence → repeat debridement + catheter removal + stop nivolumab | Improved | Severe thrombocytopenia |  |
| Lobo & Luna, 2021^(32)^ | M | 25 | Healthy; tattoo (4 weeks earlier, grey ink) | Left lower leg tattoo area | ~2 months | Culture (AFB), susceptibility, biopsy (culture negative, histology granulomatous) | Not specified | Ciprofloxacin + TMP‑SMX oral, 3 months | Cured | Liver function abnormality |  |
| Niitsu et al., 2021^(33)^ | M | 77 | Smoker, COPD, angina, post‑lung cancer surgery; immunocompetent | Pleural cavity (right pleural effusion) | 8 months (post-op; posthumous diagnosis via WGS) | Smear (AFB positive), routine culture (resembled M. fortuitum), AccuProbe, COBAS AMPLICOR, DNA‑DNA hybridization (all failed); WGS | Levofloxacin, imipenem/cilastatin (unspecified days) | Chest drainage + levofloxacin + imipenem/cilastatin (incomplete) | Died (aspiration pneumonia, CO₂ narcosis) | Death |  |
| Turuk et al., 2021^(13)^ | F | 66 | Laparoscopic cholecystectomy 1.5 years earlier; no diabetes | Abdomen (epigastric port site sinus) | ~18 months | Histopathology (non‑caseating granuloma), LJ culture (4d growth), MPT‑64 negative, line probe assay (LPA), susceptibility | Multiple broad‑spectrum antibiotics (including meropenem, vancomycin) for 1.5 y, ineffective | Ofloxacin + doxycycline oral, 2 months + sinus excision | Cured | None |  |
| Yamaguchi et al., 2021^(7)^ | F | 5y | Acute encephalopathy with adrenal insufficiency (physiologic steroids), hospitalized for acute pancreatitis | Bacteremia → right ankle subcutaneous abscess | ~2–2.5 months | Blood culture (148.2h), MALDI‑TOF MS (initially misidentified as Dermatophilus congolensis), 16S rRNA, rpoB sequencing, susceptibility | No specific empirical anti‑NTM therapy (first positive blood culture not treated) | Initial TMP‑SMX + tosufloxacin → linezolid + tosufloxacin → due to pancreatitis relapse changed to IV ciprofloxacin + imipenem/cilastatin (14 weeks); total 6 months | Cured | None |  |
| Caravedo Martinez & Blanton, 2020^(34)^ | M | 66 | Bilateral knee arthroplasty (left revised 2015) | Left prosthetic joint infection | ~6 months | AFB culture, susceptibility | Ceftriaxone 6 weeks, doxycycline, ciprofloxacin + TMP‑SMX (several days to 1 week) | Hardware removal + spacer → IV amikacin + imipenem 2 months → reimplantation → oral ciprofloxacin + TMP‑SMX 1 year | Cured | Post‑thoracotomy multiloculated left pleural effusion |  |
| Park et al., 2020^(35)^ | M | 48 | Healthy; tattoo (Philippines, 5 weeks earlier) | Left arm tattoo shaded areas | ~0.5–0.8 months | Histopathology (Fite‑Faraco AFB positive), culture (3‑4d growth, initially reported as Gram‑negative rods), MALDI‑TOF MS, susceptibility | Empiric minocycline (several days to 1 week) | Minocycline + moxifloxacin oral, 3 months | Cured | None |  |
| Singhal et al., 2020^(36)^ | M | 38 | Laparoscopic cholecystectomy | Anterior abdominal wall multiple draining sinuses | ~30 months | Ziehl‑Neelsen stain, LJ culture (3d), MALDI‑TOF MS, susceptibility, MRI | Clarithromycin + amikacin (unspecified); anti‑TB therapy 6 months | Sinus tract excision + amikacin + levofloxacin + linezolid + cotrimoxazole (6 months) | Improved | Lost to follow‑up |  |
| Hirabayashi et al., 2019^(37)^ | F | 68 | Rheumatoid arthritis, on prednisone, azathioprine, certolizumab (immunosuppressed) | Pleural cavity (bilateral pneumonia with right pleural effusion) | ~0.8–1.0 months | Ziehl‑Neelsen (AFB positive), DNA‑DNA hybridization (failed), MALDI‑TOF MS (failed), Middlebrook 7H11C culture (two colony types), repeat MALDI‑TOF MS + hsp65/rpoB sequencing (M. fortuitum + M. mageritense) | Not clearly specified | Imipenem/cilastatin + minocycline + levofloxacin (8 weeks), then minocycline + levofloxacin (4 months); total 6 months | Cured | None |  |
| Oiwa et al., 2018^(38)^ | M | 70 | Laparoscopic cholecystectomy | Abdominal multiple subcutaneous abscesses (80 nodules) | ~12 months | Culture (3d), MALDI‑TOF MS, 16S rRNA sequencing | Clarithromycin + levofloxacin + minocycline (~1 wk) | Empirical triple therapy (then stopped clarithromycin) → levofloxacin + minocycline, total 9 months | Cured | None |  |
| Tützer et al., 2018^(39)^ | F | 40 | ASD repair, autologous pericardial patch, left atrial pressure catheter | Infective endocarditis (tricuspid valve vegetation, catheter tip) | ~3 months | Blood culture (48h), Kinyoun stain, MALDI‑TOF MS (20 min), 16S rRNA sequencing | Vancomycin + amikacin (26d); meropenem + ciprofloxacin (several days) | Meropenem + ciprofloxacin IV 10 weeks  → oral ciprofloxacin + TMP‑SMX; splenectomy; total 10 weeks IV + oral | Cured | None |  |
| Fukunaga et al., 2016^(10)^ | F | 59 | Brugada syndrome, ICD for 8 years, multiple surgeries | Left chest wall ICD pocket, leads, bloodstream | >18 months (recurrent infection >1.5 y; final admission identified at 5 d) | Gram stain (misidentified as Corynebacterium), AFB stain, 16S rRNA PCR & sequencing, susceptibility | Vancomycin (several days to 1 week) | Complete lead extraction + levofloxacin + amikacin + rifampin (changed to ciprofloxacin + clarithromycin due to thrombocytopenia); total 1 y | Cured | TMP‑SMX‑induced generalized drug eruption |  |
| McMullen et al., 2015^(40)^ | F | 47 | Mechanical aortic valve replacement 6 months earlier | Prosthetic valve endocarditis, bacteremia | ~1.3–1.5 months | Blood culture (100h), Kinyoun stain, rpoB sequencing, MALDI‑TOF MS | Cefepime + rifampin + vancomycin + gentamicin (several days) | Initial: amikacin + moxifloxacin + azithromycin + imipenem → after susceptibility: doxycycline + moxifloxacin + imipenem; valve replacement at 6 weeks; oral therapy total duration not specified | Cured | Residual left hemiplegia |  |
| Muñoz‑Sanz et al., 2013^(41)^ | F | 39 | Intrathecal catheter (epidural analgesia) | Meningitis | ~2–3 months | Löwenstein culture, phenotypic identification, hsp65 PCR‑RFLP, 16S rRNA sequencing | Vancomycin + gentamicin 14d; four‑drug anti‑TB 12d | Linezolid + doxycycline + moxifloxacin (IV) → switched to oral TMP‑SMX; total 1 y | Cured | None |  |
| Huth et al., 2011^(5)^ | F | 54 | SLE, lupus nephritis, RA, hepatitis C; long‑term prednisone 15 mg/d | Lungs, pleura | ~5 months | Multiple biopsies, Fite stain (AFB positive), culture, PCR‑RFLP (hsp65), rpoB sequencing, susceptibility | Multiple antibiotics and corticosteroids (ineffective) | Imipenem + amikacin (IV) → oral linezolid + TMP‑SMX; total duration not specified, imaging resolution at 4 months | Cured | None |  |
| Ali et al., 2007^(4)^ | F | 26 | Central venous catheter (hyperemesis gravidarum) | Catheter‑related bloodstream infection | ~1.0 month | Anaerobic blood culture (103h), modified acid‑fast stain, DNA sequencing | None | IV TMP‑SMX 2 weeks+ catheter removal | Cured | None |  |
| Gira et al., 2004 (patient 1)^(42)^ | F | 43 | Healthy; footbath at nail salon (shaved legs) | Right leg pretibial (4×5 cm nodule) | ~3 months | Routine bacterial culture (3d growth), AFB stain, 65‑kDa hsp PCR‑RFLP, mycolic acid HPLC, susceptibility | Outside oral/topical antibiotics + corticosteroids (3 months) | TMP‑SMX + levofloxacin oral, 3 months | Cured | None |  |
| Gira et al., 2004 (patient 2)^(42)^ | F | 56 | Healthy; same nail salon footbath | Right leg pretibial (three ulcerated nodules) | ~8 months | Same as above | Multiple oral/topical antibiotics + corticosteroids (8 months) | Gatifloxacin oral, 2 months | Cured | None |  |

AFB = acid-fast bacilli; CKD = chronic kidney disease; COPD = chronic obstructive pulmonary disease; DIEP = deep inferior epigastric perforator flap; ESRD = end-stage renal disease; ICD = implantable cardioverter defibrillator; LAMP = loop-mediated isothermal amplification; LJ = Löwenstein-Jensen medium; MALDI‑TOF MS = matrix-assisted laser desorption/ionization time-of-flight mass spectrometry; MMF = mycophenolate mofetil; NPWT = negative pressure wound therapy; PD = peritoneal dialysis; TMP‑SMX = trimethoprim-sulfamethoxazole; TURBT = transurethral resection of bladder tumor; WGS = whole-genome sequencing.
